# *In Utero* Exposures, Infant Growth, and DNA Methylation of Repetitive Elements and Developmentally Related Genes in Human Placenta

**DOI:** 10.1289/ehp.1103927

**Published:** 2011-10-17

**Authors:** Charlotte S. Wilhelm-Benartzi, E. Andres Houseman, Matthew A. Maccani, Graham M. Poage, Devin C. Koestler, Scott M. Langevin, Luc A. Gagne, Carolyn E. Banister, James F. Padbury, Carmen J. Marsit

**Affiliations:** 1 Department of Epidemiology, Center for Environmental Health and Technology,; 2 Department of Pathology and Laboratory Medicine, and; 3 Department of Molecular Pharmacology and Physiology, Brown University, Providence, Rhode Island, USA; 4 Department of Pediatrics, Women and Infants Hospital, Providence, Rhode Island, USA; 5 Department of Pharmacology and Toxicology, Dartmouth Medical School, Hanover, New Hampshire, USA

**Keywords:** birth weight, epigenetics, fetal programming, *in utero* exposures, placenta, retrotransposon

## Abstract

Background: Fetal programming describes the theory linking environmental conditions during embryonic and fetal development with risk of diseases later in life. Environmental insults *in utero* may lead to changes in epigenetic mechanisms potentially affecting fetal development.

Objectives: We examined associations between *in utero* exposures, infant growth, and methylation of repetitive elements and gene-associated DNA in human term placenta tissue samples.

Methods: Placental tissues and associated demographic and clinical data were obtained from subjects delivering at Women and Infants Hospital in Providence, Rhode Island (USA). Methylation levels of long interspersed nuclear element-1 (LINE-1) and the Alu element AluYb8 were determined in 380 placental samples from term deliveries using bisulfite pyrosequencing. Genomewide DNA methylation profiles were obtained in a subset of 184 samples using the Illumina Infinium HumanMethylation27 BeadArray. Multiple linear regression, model-based clustering methods, and gene set enrichment analysis examined the association between birth weight percentile, demographic variables, and repetitive element methylation and gene-associated CpG locus methylation.

Results: LINE-1 and AluYb8 methylation levels were found to be significantly positively associated with birth weight percentile (*p* = 0.01 and *p* < 0.0001, respectively) and were found to differ significantly among infants exposed to tobacco smoke and alcohol. Increased placental AluYb8 methylation was positively associated with average methylation among CpG loci found in polycomb group target genes; developmentally related transcription factor binding sites were overrepresented for differentially methylated loci associated with both elements.

Conclusions: Our results suggest that repetitive element methylation markers, most notably AluYb8 methylation, may be susceptible to epigenetic alterations resulting from the intrauterine environment and play a critical role in mediating placenta function, and may ultimately inform on the developmental basis of health and disease.

The importance of environmental exposures during intrauterine development on health throughout the life course is now well recognized in the epidemiologic literature (Barker 2004). The molecular mechanisms that underlie these observations remain unclear, although recent work suggests that alterations to placental function may lead to altered fetal development and programming, likely playing a critical role in mediating these associations (Higgins et al. 2011; Lester and Padbury 2009). Throughout *in utero* development, the placenta, through the production of various enzymes and hormones, plays an important role in controlling growth and development through the transfer of nutrients and waste and in protecting the fetus from many xenobiotic insults (Robins et al. 2011). Recent work has demonstrated that placental genetic and epigenetic profiles may possibly serve as markers (Filiberto et al. 2011; Sood et al. 2006) of the intrauterine and extrauterine environment (Nelissen et al. 2011).

DNA methylation is a key mode of epigenetic regulation, which can lead to silencing of genomic regions. DNA methylation patterns are essential for the growth and maintenance of tissue-specific expression profiles in different cell types during development, and these patterns become set during *in utero* development (Hajkova et al. 2002; Jaenisch 1997; Rougier et al. 1998). This is true both in the fetus itself and in the placenta, where changes to the appropriate methylation patterning have been linked to adverse placental morphology and birth outcomes (Serman et al. 2007; Sinclair et al. 2007).

Studies examining the impact of environmental exposures on DNA methylation have often focused on the global extent of 5-methylcytosine, using various approaches (Einstein et al. 2010; Pavanello et al. 2009), including the examination of repetitive elements as surrogate markers of global methylation (Choi et al. 2009; Lupski 2010; Weisenberger et al. 2005). These DNA repetitive elements are made up of interspersed and tandem repeats and comprise at least half of the human genome (Zamudio and Bourc’his 2010). Specifically, interspersed repeats are composed of retrotransposable elements such as long interspersed nuclear elements (LINEs) and short interspersed nuclear elements (SINEs). The LINE-1 subclass is the most common LINE, representing about 20% of the total human genome, whereas the most abundant type of SINE is the Alu element, as exemplified by AluYb8; together *LINE* and *Alu* sequences make up nearly one-third of the genome (Choi et al. 2009; Zamudio and Bourc’his 2010). The methylation status of these elements may represent a passive dosimeter by being altered by various exposures, although differences in the underlying structure of these elements and the unclear function of methylation of these regions suggest that their alteration and their potential relevance need to be more carefully examined.

Thus, in this study we examined how the intrauterine environment is related to the methylation status of LINE-1 and Alu regions (specifically *LINE-1* and *AluYb8*, respectively) in a functionally relevant fetal tissue, the placenta. Further, we aimed to explore whether alterations to the methylation of these regions are associated with methylation of gene-associated loci using a genomewide bioinformatic approach, in order to provide clues about the potential importance of these alterations.

## Methods

*Study population.* Residual placental tissues were obtained from 479 patients delivering at the Women and Infants Hospital in Providence, Rhode Island (USA), from September 2008 through September 2009. This hospital performs approximately 85% of all obstetrical deliveries in Providence County (the largest population center in Rhode Island) and approximately 74% of all deliveries in Rhode Island. Study eligibility criteria for mothers included healthy mothers ranging from 18 to 40 years of age, with no history of gestational diabetes, psychological disorders, or genetic disorders. Eligibility criteria for infants included viable infants of term births (born > 37 weeks of gestation) with no known genetic disorders or life-threatening illnesses. We selected infants considered small for gestational age (SGA; < 10th percentile of birth weight) and matched non-SGA infants to these for infant sex, maternal age (± 3 years), and gestational age (± 2 days). For a description of placental tissue use, DNA extraction, and DNA purification procedures, see Supplemental Material, Supplementary Text 1 (http://dx.doi.org/10.1289/ehp.1103927). All procedures were approved by the appropriate institutional review boards at Women and Infants Hospital and at Brown University, and participants were exempted from informed consent requirements.

*Covariate data.* All covariate data were collected from a structured inpatient medical record review. Birth weight percentile was calculated using the Fenton growth chart from gestational age and birth weight in grams (Fenton 2003), allowing us to account for gestational age in our birth weight metric and to create a normalized distribution for birth weight through birth weight percentile. Birth weight percentile was stratified into three clinically meaningful categories to create birth weight status: SGA babies, defined as babies whose birth weight lies below the 10th percentile for that gestational age; appropriate for gestational age (AGA) babies, defined as babies whose birth weight lies above the 10th percentile for that gestational age and below the 90th percentile for that gestational age; and large for gestational age (LGA) babies, defined as babies whose birth weight lies above the 90th percentile for that gestational age.

*DNA methylation analysis.* Repetitive element DNA methylation was determined by bisulfite pyrosequencing of the LINE-1 and the AluYb8 repetitive elements as previously described (Bollati et al. 2007; Choi et al. 2009). Methylation extent was calculated as the mean LINE-1 methylation of four positions in the element and mean AluYb8 methylation of five positions in the element. Pyrosequencing of these repeat elements was successful in 380 samples; we noted no significant differences in the demographics or clinical characteristics of these samples compared with the larger population.

To examine gene-related CpG methylation on a subset of the samples (*n* = 184), methylation was measured at 27,578 CpG loci using the Infinium HumanMethylation27 BeadArray (Illumina, San Diego, CA). The microarrays were processed at the University of California–San Francisco Institute for Human Genetics and Genomics Core Facility, following standard protocols. The methylation status for each individual CpG locus was calculated as the ratio of fluorescent signals:

β = max(*M*,0)_/_[max(*M*,0) + max(*U*,0) + 100],

using the average probe intensity for the methylated (*M*) and unmethylated (*U*) alleles. Ratios range from 0 to 1: β = 1 indicates complete methylation and β = 0 represents no methylation. The data were then assembled using BeadStudio methylation software (version 1.9.0; Illumina), without normalization, per the manufacturer’s instructions. We used array control probes to assess the quality of our samples and evaluate potential problems such as poor bisulfite conversion or color-specific issues for each array. Mahalanobis distance was used to screen outliers, and all CpG loci on X and Y chromosomes were excluded from the analysis, to avoid sex-specific methylation bias, leaving a final 26,486 autosomal CpG loci representing 13,890 unique genes (Poage et al. 2011). We and others have previously demonstrated that methylation of CpG loci detected through BeadArray platforms can be reliably replicated using alternative detection techniques including pyrosequencing, MassARRAY analysis, and quantitative methylation-specific polymerase chain reaction (Ang et al. 2010; Christensen et al. 2010; Lin et al. 2010; Marsit et al. 2010; Poage et al. 2010, 2011; Wolff et al. 2010).

*Statistical methods.* Univariate associations of exposures and demographics with birth weight percentile status or with LINE-1 and AluYb8 extent were examined using Kruskal–Wallis tests for continuous covariates and chi-square tests for categorical covariates. A Pearson correlation was used to examine the correlation between LINE-1 and AluYb8 extents. Multiple linear regression was then used to evaluate the association between LINE-1 or AluYb8 methylation levels and continuous birth weight percentile. All models were adjusted for infant sex and maternal age, body mass index (BMI) before pregnancy, ethnicity (non-Caucasian/Caucasian), and alcohol, tobacco, and prenatal vitamin use during pregnancy (all yes/no). To check the linearity assumption for the regression models, we plotted residuals by birth weight percentile for all models and found no evidence for systematic nonlinearity.

To examine how repetitive element methylation was related to gene-associated methylation, we restricted our analysis to placentas for which we had both repetitive element and gene-associated (array) methylation data for the 26,486 CpG autosomal loci (*n* = 184). Using a data-driven recursively partitioned mixture model (RPMM), these CpG loci were clustered into 16 methylation classes based on like methylation pattern obtained from the array results, pruning the model after four splits to maintain power (Houseman et al. 2008). For each subject, 16 corresponding aggregate methylation values were obtained by averaging together average methylation β-values from all CpGs within each class. RPMM classes were labeled using a series of numbers, ranging from 1 to 16 (with class 1 having the lowest methylation level and class 16 having the highest methylation level). RPMM was selected for this analysis because we have demonstrated that RPMM provides more consistent clustering and more robustness in the selection of the number of classes because of its hierarchical presentation of classes, compared with metric hierarchical clustering, for this data set [see Supplemental Material, Supplementary Text 2 (http://dx.doi.org/10.1289/ehp.1103927)]. An alternative, attribute-driven clustering scheme for the 26,486 autosomal CpG loci was employed by grouping CpGs by epigenetically relevant bioinformatic attributes: CpG island (CGI) status (Takai and Jones 2002), polycomb group (PcG) target status of the associated gene [i.e., gene was described as a PcG target in at least one of Bracken et al. (2006), Lee et al. (2006), Schlesinger et al. (2007), and/or Squazzo et al. (2006)], presence within 1 kb of at least one of 258 computationally predicted transcription factor binding site (TFBS) sequences obtained from the tfbsConsSites track of the University of California–Santa Cruz (UCSC) Genomes Browser site (TFBS *z*-score > 2) (Kent et al. 2002), and localization within each of the following classes of repetitive element as defined by the Repeatmasker track of Genomes Browser: Alu, LINE-1, LINE-2, and mammalian wide-interspersed repeat elements (MIRs). This bioinformatic classification resulted in 41 distinct bioinformatic classes containing at least one CpG (see Supplemental Material, Table 2). For each subject, 41 corresponding aggregate methylation values were obtained by averaging together average β-values from all CpGs within the class.

**Table 1:** Associations between infant growth and repetitive element methylation.

Covariate (*n* = 380)	Linear regression effect estimate (95% CI)	*p*-Value
Mean LINE-1 (per 10%)		9.7 (2.9, 16.6)		0.01
Infant sex		12.81 (6.54, 19.09)		< 0.0001
White maternal ethnicity		7.06 (0.59, 13.53)		0.03
Mean AluYb8 (per 10%)		14.5 (4.9, 24.0)		< 0.0001
Infant sex		12.05 (5.76, 18.33)		< 0.0001
White maternal ethnicity		6.91 (0.46, 13.36)		0.04
All models were also adjusted for maternal age, BMI before pregnancy, tobacco, alcohol, and prenatal vitamin use during pregnancy. Infant sex uses female sex as the referent.				

**Table 2:** Repeat element methylation markers grouped by *in utero* exposures.

Group mean ± SD				
Exposure	*n*	LINE-1	AluYb8	Array-based methylation
Sex								
Male		190		51.7 ± 4.4		65.3 ± 3.3		0.238 ± 0.012
Female		190		51.7 ± 4.9		64.6 ± 3.3		0.237 ± 0.013
*p*-Value				0.90		0.04		0.28
Maternal ethnicity								
Caucasian		217		51.4 ± 4.5		64.9 ± 3.3		0.239 ± 0.013
Non-Caucasian		163		52.0 ± 4.8		65.1 ± 3.3		0.236 ± 0.011
*p*-Value				0.21		0.47		0.17
Maternal tobacco use during pregnancy								
Yes		36		52.9 ± 4.7		66.2 ± 2.3		0.240 ± 0.011
No		343		51.6 ± 4.6		64.8 ± 3.4		0.237 ± 0.012
*p*-Value				0.10		< 0.01		0.31
Maternal alcohol use during pregnancy								
Yes		3		55.0 ± 1.0		66.0 ± 3.4		0.230 ± 0.001
No		377		51.7 ± 4.6		65.0 ± 3.3		0.238 ± 0.012
*p*-Value				0.02		0.66		0.51
Maternal prenatal vitamin use during pregnancy								
Yes		314		51.8 ± 4.6		65.0 ± 3.3		0.238 ± 0.012
No		66		51.3 ± 4.7		64.6 ± 3.4		0.238 ± 0.014
*p*-Value				0.50		0.29		0.80
*p*-Values obtained from Kruskal–Wallis tests.								

We used linear regression to determine the association between methylation of LINE-1 or AluYb8 and the aggregate methylation values for each of the 16 methylation classes determined by RPMM and the 41 bioinformatic classes. Omnibus tests for the overall association between each repetitive methylation and class methylation were obtained by permutation test, using a test analogous to a Kolmogorov–Smirnov test. Specifically, a test statistic was constructed as the maximum absolute *t*-statistic for the appropriate coefficient from the regression model, where the maximum was computed over the 16 or 41 individual regression models. The corresponding null distribution, signifying a lack of association between repetitive element methylation and autosomal CpG methylation, was obtained by randomly permuting each repetitive element methylation marker with respect to aggregate methylation values of the RPMM and bioinformatic classes. Ten thousand permutations were used, and a hypothesized association was considered significant at the *p* ≤ 0.05 level.

To clarify the TFBSs overrepresented among loci whose methylation was associated with either LINE-1 or AluYb8 methylation, gene set enrichment analysis (GSEA) (Subramanian et al. 2005) was used. Across binding sites of transcription factors overrepresented within 1 kb of loci, we compared the distribution of statistics representing the association between CpG-specific average β-value and each repetitive element methylation markers. All statistical analyses were performed using the R statistical package (version 2.10; http://www.r-project.org), and the R function was made available on the GSEA website, using permutation tests as described by Subramanian et al. (2005).

## Results

*Population characteristics.* The characteristics of subjects used in these analyses (*n* = 380) are shown in Supplemental Material, Table 1 (http://dx.doi.org/10.1289/ehp.1103927). Because subjects were selected based on SGA status and matched to non-SGA infants on sex, gestational age, and maternal age, we observed no significant differences in those characteristics among birth weight groups (SGA, AGA, LGA). Significant differences were noted between groups for maternal ethnicity and maternal tobacco smoking during pregnancy, with a greater proportion of non-Caucasian infants (*p* = 0.01) and infants with exposure to tobacco smoke in the SGA category (*p* = 0.03). The vast majority of mothers reported prenatal vitamin use; only three subjects reported alcohol consumption during pregnancy.

*DNA methylation and infant growth.* Mean LINE-1 and AluYb8 methylation levels differed significantly by birth weight status [see Supplemental Material, Table 1 (http://dx.doi.org/10.1289/ehp.1103927); *p* < 0.0001]. Mean LINE-1 and AluYb8 methylation levels were also found to be weakly to moderately correlated, with a Pearson correlation of 0.29 [95% confidence interval (CI): 0.20, 0.38]. To examine whether “global methylation” represented as an average of gene-associated methylation identified in a genomewide manner was associated with infant growth, we tested for an association between birth weight percentile and mean methylation across the 26,486 autosomal loci measured using the Illumina Infinium array in a subset of 184 placenta samples. Mean methylation across the 26,486 autosomal CpG loci was not significantly associated with the birth weight groups (see Supplemental Material, Table 1). There were no significant differences in the distributions of the demographic characteristics of this subsample and the larger population examined for LINE-1 and AluYb8 methylation extent (data not shown).

To further evaluate the association between infant birth weight and repetitive element methylation identified in the univariate analyses, controlled for confounders, we used multiple linear regression. These models demonstrate that with a 10% increase in LINE-1 mean methylation levels, birth weight percentile significantly increased by 9.7 (95% CI: 2.9, 16.6), and with a 10% increase in AluYb8 mean methylation levels, birth weight percentile significantly increased by 14.5 (95% CI: 4.9, 24.0) (Table 1). Both models were adjusted for infant sex and maternal age, BMI, ethnicity, and tobacco, alcohol and prenatal vitamin use during pregnancy. Infant sex and maternal ethnicity were also significant predictors of birth weight percentile (Table 1); all other covariates were not significant.

*DNA methylation and* in utero *exposures.* Table 2 presents the results of our exploration of the differences in the measures of DNA methylation by various *in utero* exposures. Only AluYb8 methylation levels differed by infant sex (*p* = 0.04), with male infants having slightly higher mean AluYb8 methylation (mean ± SD = 65.3 ± 3.3) than did female infants (64.6 ± 3.3). Furthermore, we found that mean AluYb8 levels differed significantly by maternal tobacco use during pregnancy (*p* < 0.01), whereas mean LINE-1 levels significantly differed only by maternal alcohol use during pregnancy (*p* = 0.02; Table 2). Array-based mean methylation across the 26,486 CpG loci did not differ by any of the *in utero* exposures explored.

*Relationships between repetitive element and gene associated methylation.* To investigate the relationship between repeat element methylation and gene-associated methylation, we clustered the 26,486 autosomal CpG loci interrogated on the Infinium HumanMethylation27 BeadArray into 16 methylation classes based on their methylation pattern using an RPMM [for the heat map of the 16 classes, see Supplemental Material, Figure 1 (http://dx.doi.org/10.1289/ehp.1103927)]. This data-driven clustering allowed us to define stable classes of CpG loci demonstrating similar patterns of methylation, which we used to examine associations between methylation of these classes of CpGs and LINE-1 or AluYb8 methylation. By clustering, we hoped to uncover biologically meaningful groups of CpG loci, with similar methylation patterns, which may share biological characteristics such as comparable functionality in certain developmental pathways or location in similar genomic regions.

**Figure 1:**
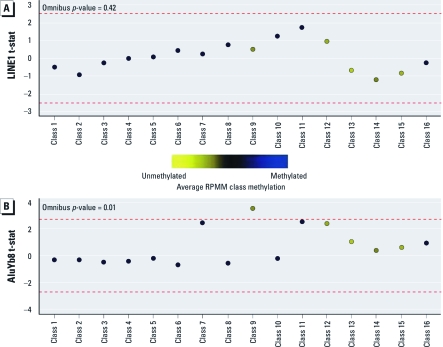
Association of LINE-1 (*A*) and AluYb8 (*B*) methylation with mean RPMM class methylation. The colored dots indicate the degree of average CpG class methylation, as indicated by the key. The red dashed lines represent the null limits for the permutation distribution of regression coefficient *t*-statistics (t-stat), adjusted for multiple comparisons.

The association between LINE-1 or AluYb8 methylation and average methylation within the RPMM-derived classes for each of the 184 individuals in the study is presented in Figure 1. There was an overall significant relationship between AluYb8 methylation and RPMM class methylation (*p* = 0.01); no association was observed between LINE-1 and RPMM class methylation. When considering class-specific relationships, a positive association was noted between AluYb8 methylation and methylation of CpGs encompassing the low to intermediately methylated class 9 (Figure 1). Additionally, classes 7, 11, and 12 were marginally positively associated with AluYb8 methylation levels, with CpGs exhibiting low to intermediate methylation.

**Figure 2:**
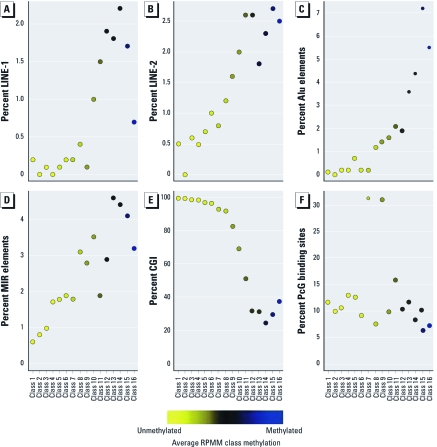
Frequency of sequence features associated with RPMM class CpG loci: percentage of CpG loci found in LINE-1 (*A*), LINE-2 (*B*), Alu (*C*), and MIR (*D*), within a CGI (*E*), or having the associated gene considered a PcG protein target gene (*F*). The colored dots indicate the degree of average class methylation.

To examine similarities in the genomic context of the CpGs making up these data-driven RPMM classes, we examined the proportion of CpG loci associated with specific DNA sequence features within each class, including location within a repetitive element (LINE-1, LINE-2, Alu, or MIR), within a CGI, or having the associated gene considered a PcG protein target gene (Figure 2). As expected, a greater proportion of CpGs in the relatively methylated (rightmost) classes 9–12 are localized in repetitive elements (Figure 2A–D). Conversely, a high proportion of CpGs in the relatively unmethylated classes 1–6 and 8 are in CGIs (Figure 2E). There was also interclass variation in the frequency of CpGs association with PcG target genes, with classes 7 and 9 in particular having a much higher proportion than the others (Figure 2F). Class 9, which was associated with AluYb8 methylation extent, contained a high proportion of CpG loci located within CGIs (81%) and whose genes are considered PcG target genes (32%).

**Figure 3:**
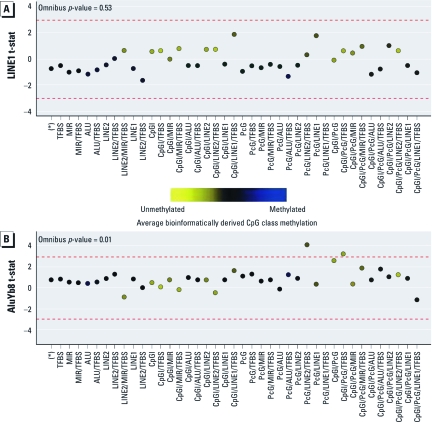
Association of LINE-1 (*A*) and AluYb8 (*B*) methylation with bioinformatically derived CpG class methylation. The colored dots indicate the degree of average class methylation. The red dashed lines represent the null limits for the permutation distribution of regression coefficient *t*-statistics (t-stat), adjusted for multiple comparisons.

Motivated by the variation in sequence features (bioinformatic attributes) observed between RPMM-based classes, we next performed a bioinformatically informed clustering of CpG loci into classes, grouping CpGs according to their sequence context: presence within a CGI, LINE-1, LINE-2, Alu, or MIR element, or PcG target gene and proximity (≤ 1,000 bases) to any TFBS. This resulted in 41 distinct bioinformatically derived classes, containing at least one CpG, based on bioinformatic attributes [for the distribution of CpG loci by bioinformatically derived class, see Supplemental Material, Table 2 (http://dx.doi.org/10.1289/ehp.1103927)]. The association between LINE-1 and AluYb8 repetitive element methylation and aggregate methylation values of the autosomal CpG loci within each of the 41 bioinformatic classes is depicted in Figure 3. There was an overall significant relationship between AluYb8 methylation extent and bioinformatically derived CpG class methylation (*p* < 0.0001) but not between LINE-1 methylation and bioinformatically derived CpG class methylation. When considering class-specific relationships, there was a positive association between AluYb8 methylation levels and mean methylation across loci allocated within a PcG target gene and LINE-2 element and proximal to a TFBS (class PcG/LINE-2/TFBS) and loci located within a CGI and PcG target gene and proximal to a TFBS (class CpG/PcG/TFBS).

*GSEA analysis.* To expand on the bioinformatically informed analysis, which suggested that methylation of CpG loci proximal to a TFBS were associated with AluYb8 methylation, we performed a GSEA using specific TFBS, obtained from the UCSC Genomes Browser. Specifically, each of the 26,486 autosomal CpG loci are simultaneously evaluated for their association with LINE-1 or AluYb8 methylation extent and the genomic locations or TFBS that are overrepresented within 1 kb of the loci [for the GSEA results, see Supplemental Material, Figure 2 (http://dx.doi.org/10.1289/ehp.1103927)]. Only three TFBS genes were overrepresented among loci whose methylation was associated with LINE-1 methylation: *MEF2*, *TBX1*, and *POU2F1*. For those overrepresented among loci associated with AluYb8 methylation, there were a number of developmentally related TFBS genes (*CUX1*, *CHX10*, *PAX4*, *PPARG*, *TAL1/ITF2* heterodimer, *HAND1*, MYOD, OCT), as well as those involved in cell processes (*ACTR3B*, *S8*), gene expression (*NFE2L*), lipid/sterol homeostasis (*NR3C1*), and immune modulation (*GATA1*). Only *POU2F1* was found to be overrepresented among loci associated with both LINE-1 and AluYb8 methylation.

## Discussion

In this study, we have identified significant associations between infant growth, *in utero* exposures, and repetitive element methylation in placental tissue. Moreover, by examining the relationship between repetitive element DNA methylation and genomewide (array) gene-associated methylation, this work suggests potential epigenetic mechanisms underlying changes to placental function that may lead to altered fetal development and programming. In addition, we have identified particular regions of the genome susceptible to alterations by the intrauterine environment.

Overall, AluYb8 had higher DNA methylation levels than did LINE-1, which may be due to the lower degree of C-to-T transition mutations owing to the younger age of AluYb8 elements (Choi et al. 2009). AluYb8 levels were found to marginally differ by infant sex, which is consistent with the previous literature in that repetitive element methylation markers in blood may vary by sex (Cash et al. 2011; Wilhelm et al. 2010). We found that birth weight percentile levels increased by about 10 and 15 points, respectively, with a 10% increase in LINE-1 or AluYb8 methylation levels, suggesting a strong association of repetitive element methylation markers with fetal growth. However, because LINE-1 and AluYb8 methylation levels have a moderate correlation, it is possible that these two repetitive element methylation markers may explain both similar and different sources of variation in infant growth. Our analysis of the relationship between the extent of methylation at these elements and genomewide gene-associated methylation was aimed to better characterize the similarities and differences in the potential functional importance of these markers.

The approach to examining the relationship between repetitive element methylation and gene-associated methylation was not designed to assess specific loci or genes associated with LINE-1 or AluYb8 methylation, but rather to evaluate whether methylation of particular groups of CpG loci with like methylation patterns or similar bioinformatic attributes were coordinately associated with LINE-1 or AluYb8 methylation, because this could inform on the process responsible for the variation between the two. Based on our data-driven RPMM approach, we observed that AluYb8 methylation was significantly and positively correlated with methylation at CpG loci in an intermediately methylated class containing loci that were located predominantly within CGIs and associated with PcG target genes. This was further substantiated in our bioinformatically informed classification analysis, because we identified a significant association between AluYb8 methylation and methylation of a group of loci defined by their position in a CGI, PcG target gene, and a TFBS, as well CpGs defined by association with a PcG target gene and LINE-2 element and proximal to a TFBS. We did not observe an overall association between any attribute-defined gene-associated class methylation and LINE-1 methylation levels. This is in contrast to a previous study involving 12 fetal cord blood samples, which reported a significant correlation between LINE-1 methylation and methylation of specific loci residing mostly in CGIs (Fryer et al. 2011). This disparity may reflect tissue-specific differences in these relationships, as well as the larger and therefore more precise nature of our examination. PcG targets are a family of developmentally related genes that can remodel chromatin and are responsible for silencing *HOX* genes, which are critical in embryonic development as well as potentially hundreds of other genes involved in cellular differentiation (Kirmizis et al. 2004; Simon and Kingston 2009). We observed that increasing AluYb8 methylation was associated with greater extents of methylation at CpG loci located in a PcG target, and thus a change in the extent of methylation of AluYb8 may represent an alteration in the methylation of these key loci important in tissue development and differentiation.

Both bioinformatically informed CpG classes that were significantly associated with AluYb8 methylation contained loci that are proximal to TFBSs. Upon further exploration through GSEA, we observed an overrepresentation of several TFBSs for transcription factors critical in development, cellular processes, immune modulation, lipid or sterol homeostasis, and gene expression near loci associated with AluYb8, as well as a few developmental and cellular proliferation pathways for LINE-1. Specifically, *POU2F1* binding sites were overrepresented among loci associated with both LINE-1 and AluYb8 extent. This transcription factor plays a role in the control of a wide range of genes, including small nuclear RNAs, immunoglobulins, histone H2B, and the glucocorticoid receptor, and thus has a central role in controlling various functions, including those of immunomodulation and steroid homeostasis in the placenta (Schonemann et al. 1998). Taken together, these results suggest that alterations to LINE-1 or AluYb8 methylation may act as a marker for key changes to the extent of methylation at loci targeted by important transcription factors for normal development and placenta processes necessary for appropriate growth.

When looking at each repetitive element methylation marker grouped by various *in utero* exposures, we found that mean AluYb8 methylation levels were significantly different by maternal tobacco use during pregnancy, similar to results from another group that found significantly different levels of cytosine methylation by smoking status (Moore et al. 2008). Furthermore, mean LINE-1 methylation levels were significantly different by maternal alcohol use during pregnancy; however, limited numbers may affect our precision, so this result should be confirmed in further analyses. This study was limited in its assessment of environmental exposures because of the low number of exposed individuals and low power, making this part of our analysis largely exploratory in nature. However, it will be increasingly important to examine, in larger studies, how additional specific exposures, such as lead as has been done by previous researchers (Pilsner et al. 2009), during intrauterine development affect these epigenetic markers in a more quantitative way.

Although a few other studies have correlated array-based methylation levels with repetitive element methylation markers (Fryer et al. 2011) and found at times good correlation between the two, our results indicate that array-based methylation level may not be a good predictive marker of repetitive element methylation level in human placenta tissue. Array-based mean methylation across the 26,486 CpG loci was not found to be associated with any of our *in utero* exposures, contrasting with our repetitive element methylation markers. Additionally, LINE1, AluYb8, and specific array-based methylation loci often interrogate different genetic regions; therefore, their methylation may have a differential genomic impact. Furthermore, the divergence of results observed between LINE-1 and AluYb8 reported throughout this work suggests that these repetitive element methylation markers are distinct measures and may not represent true, and potentially interchangeable, surrogates of “global” methylation. Although we found that these repetitive element indicators are weakly to moderately correlated, they have their own unique distributions of methylation values, consistent with previous comparisons between LINE-1 and Alu methylation in normal blood (Jintaridth and Mutirangura 2010). In addition, it is believed that genomic location and sequence context play a crucial role in the maintenance or loss of DNA methylation in these regions. For example, research has suggested that Alu sequences often flank CGIs (Graff et al. 1997) and therefore may play a distinct role in both repetitive element and gene-directed methylation. Differences between these markers can most likely be explained by the unequal distributions of these sequences throughout the genome, owing in some part to the evolutionary age of these two retrotransposable elements (Zamudio and Bourc’his 2010). This may therefore be affecting the number of these elements in the genome and the functional relevance of their methylation, particularly in the placenta. As our results suggest, distinct mechanisms and selective pressures are likely responsible for their alteration.

The strengths of this study include the large sample size, population-based ascertainment of placenta samples, quantitative assessment of LINE-1 and AluYb8 methylation, and the use of a well-established, high-density methylation array for methylation profiling. Modest limitations of this study include the use of data from medical records, which may have reduced the quality of covariate data used in this study. This may have been especially relevant in our measurement of maternal tobacco, alcohol, and prenatal vitamin use and could have potentially introduced a bias toward the null. Additionally, because Rhode Island is a small state representing a small geographic area, recruiting participants from this state may affect the generalizability of our findings; however, because the Women and Infants Hospital performs > 85% of all obstetrical deliveries in Providence County (the largest population center in Rhode Island), we believe this limitation is compensated for to some extent by the lack of ascertainment bias in this population-based study.

## Conclusions

Overall, we have demonstrated that altered methylation of repetitive elements in the placenta is associated with birth outcome and with specific environmental exposures encountered by the fetus. Of course, birth weight is likely a proxy for a complex interplay of underlying etiologic mechanisms and environmental factors that overall contribute to infant health at birth and beyond. Our data also suggest that these alterations may reflect underlying functional epigenetic alterations to genes important in placental growth and development. Confirmation and expansion of these findings in additional populations, with additional environmental data and more comprehensive methylation profiles as well as studies of the mechanism of these alterations, will provide additional valuable insights into the epigenetic mechanisms controlling fetal growth. These data also further the hypothesis that the intrauterine environment acting through epigenetic alteration of the placenta is a key mechanism to explain the developmental origins of health and disease and suggest that enhanced examinations of the importance of placental epigenetic variation in health outcomes should be undertaken.

## Supplemental Material

(4.3 MB) PDF

## References

[r1] Ang PW Loh M Liem N Lim PL Grieu F Vaithilingam A 2010 Comprehensive profiling of DNA methylation in colorectal cancer reveals subgroups with distinct clinicopathological and molecular features. BMC Cancer 10 227; doi:10.1186/1471-2407-10-227 [Online 21 May 2010] 20492682 PMC2880997

[r2] Barker DJ (2004). The developmental origins of adult disease.. J Am Coll Nutr.

[r3] Bollati V, Baccarelli A, Hou L, Bonzini M, Fustinoni S, Cavallo D (2007). Changes in DNA methylation patterns in subjects exposed to low-dose benzene.. Cancer Res.

[r4] Bracken AP, Dietrich N, Pasini D, Hansen KH, Helin K (2006). Genome-wide mapping of Polycomb target genes unravels their roles in cell fate transitions.. Genes Dev.

[r5] Cash HL Tao L Yuan JM Marsit CJ Houseman EA Xiang YB 2011 LINE-1 hypomethylation is associated with bladder cancer risk among non-smoking Chinese. Int J Cancer; doi:10.1002/ijc.26098 [Online 25 May 2011] PMC3208798 21445976

[r6] Choi SH, Worswick S, Byun HM, Shear T, Soussa JC, Wolff EM (2009). Changes in DNA methylation of tandem DNA repeats are different from interspersed repeats in cancer.. Int J Cancer.

[r7] Christensen BC Kelsey KT Zheng S Houseman EA Marsit CJ Wrensch MR 2010 Breast cancer DNA methylation profiles are associated with tumor size and alcohol and folate intake. PLoS Genet 6 7 e1001043; doi:10.1371/journal.pgen.1001043 [Online 29 July 2010] 20686660 PMC2912395

[r8] Einstein F Thompson RF Bhagat TD Fazzari MJ Verma A Barzilai N 2010 Cytosine methylation dysregulation in neonates following intrauterine growth restriction. PLoS One 5 1 e8887; doi:10.1371/journal.pone.0008887 [Online 26 January 2010] 20126273 PMC2811176

[r9] Fenton TR 2003 A new growth chart for preterm babies: Babson and Benda’s chart updated with recent data and a new format. BMC Pediatr 3 13; doi:10.1186/1471-2431-3-13 [Online 16 December 2003] 14678563 PMC324406

[r10] Filiberto A, Maccani M, Koestler D, Wilhelm-Benartzi C, Gagne L, Marsit C. (2011). Birthweight is associated with DNA promoter methylation of the glucocorticoid receptor in the human placenta.. Epigenetics.

[r11] Fryer AA, Emes RD, Ismail KM, Haworth KE, Mein C, Carroll WD (2011). Quantitative, high-resolution epigenetic profiling of CpG loci identifies associations with cord blood plasma homocysteine and birth weight in humans.. Epigenetics.

[r12] Graff JR, Herman JG, Myohanen S, Baylin SB, Vertino PM (1997). Mapping patterns of CpG island methylation in normal and neoplastic cells implicates both upstream and downstream regions in de novo methylation.. J Biol Chem.

[r13] Hajkova P, Erhardt S, Lane N, Haaf T, El-Maarri O, Reik W (2002). Epigenetic reprogramming in mouse primordial germ cells.. Mech Dev.

[r14] Higgins L, Greenwood SL, Wareing M, Sibley CP, Mills TA (2011). Obesity and the placenta: a consideration of nutrient exchange mechanisms in relation to aberrant fetal growth.. Placenta.

[r15] Houseman EA Christensen BC Yeh RF Marsit CJ Karagas MR Wrensch M 2008 Model-based clustering of DNA methylation array data: a recursive-partitioning algorithm for high-dimensional data arising as a mixture of beta distributions. BMC Bioinformatics 9 365; doi:10.1186/1471-2105-9-365 [Online 9 September 2008] 18782434 PMC2553421

[r16] Jaenisch R. (1997). DNA methylation and imprinting: why bother?. Trends Genet.

[r17] Jintaridth P, Mutirangura A. (2010). Distinctive patterns of age-dependent hypomethylation in interspersed repetitive sequences.. Physiol Genom.

[r18] Kent WJ, Sugnet CW, Furey TS, Roskin KM, Pringle TH, Zahler AM (2002). The human genome browser at UCSC.. Genome Res.

[r19] Kirmizis A, Bartley SM, Kuzmichev A, Margueron R, Reinberg D, Green R (2004). Silencing of human polycomb target genes is associated with methylation of histone H3 Lys 27.. Genes Dev.

[r20] Lee TI, Jenner RG, Boyer LA, Guenther MG, Levine SS, Kumar RM (2006). Control of developmental regulators by Polycomb in human embryonic stem cells.. Cell.

[r21] Lester BM, Padbury JF (2009). Third pathophysiology of prenatal cocaine exposure.. Dev Neurosci.

[r22] Lin Z, Hegarty J, Cappel J, Yu W, Chen X, Faber P (2010). Identification of disease-associated DNA methylation in intestinal tissues from patients with inflammatory bowel disease.. Clin Genet.

[r23] Lupski JR (2010). Retrotransposition and structural variation in the human genome.. Cell.

[r24] Marsit CJ Houseman EA Christensen BC Gagne L Wrensch MR Nelson HH 2010 Identification of methylated genes associated with aggressive bladder cancer. PLoS One 5 8 e12334; doi:10.1371/journal.pone.0012334 [Online 23 August 2010] 20808801 PMC2925945

[r25] Moore LE, Pfeiffer RM, Poscablo C, Real FX, Kogevinas M, Silverman D (2008). Genomic DNA hypomethylation as a biomarker for bladder cancer susceptibility in the Spanish Bladder Cancer Study: a case-control study.. Lancet Oncol.

[r26] Nelissen EC, van Montfoort AP, Dumoulin JC, Evers JL (2011). Epigenetics and the placenta.. Hum Reprod Update.

[r27] Pavanello S, Bollati V, Pesatori AC, Kapka L, Bolognesi C, Bertazzi PA (2009). Global and gene-specific promoter methylation changes are related to anti-B[a]PDE-DNA adduct levels and influence micronuclei levels in polycyclic aromatic hydrocarbon-exposed individuals.. Int J Cancer.

[r28] Pilsner JR, Hu H, Ettinger A, Sanchez BN, Wright RO, Cantonwine D (2009). Influence of prenatal lead exposure on genomic methylation of cord blood DNA.. Environ Health Perspect.

[r29] Poage GM Christensen BC Houseman EA McClean MD Wiencke JK Posner MR 2010 Genetic and epigenetic somatic alterations in head and neck squamous cell carcinomas are globally coordinated but not locally targeted. PLoS One 5 3 e9651; doi:10.1371/journal.pone.0009651 [Online 11 March 2010] 20300172 PMC2836370

[r30] Poage GM, Houseman EA, Christensen BC, Butler RA, Avissar-Whiting M, McClean MD (2011). Global hypomethylation identifies loci targeted for hypermethylation in head and neck cancer.. Clin Cancer Res.

[r31] Robins JC, Marsit CJ, Padbury JF, Sharma SS (2011). Endocrine disruptors, environmental oxygen, epigenetics and pregnancy.. Front Biosci.

[r32] Rougier N, Bourc’his D, Gomes DM, Niveleau A, Plachot M, Paldi A (1998). Chromosome methylation patterns during mammalian preimplantation development.. Genes Dev.

[r33] Schlesinger Y, Straussman R, Keshet I, Farkash S, Hecht M, Zimmerman J (2007). Polycomb-mediated methylation on Lys27 of histone H3 pre-marks genes for de novo methylation in cancer.. Nat Genet.

[r34] Schonemann MD, Ryan AK, Erkman L, McEvilly RJ, Bermingham J, Rosenfeld MG (1998). POU domain factors in neural development.. Adv Exp Med Biol.

[r35] Serman L, Vlahovic M, Sijan M, Bulic-Jakus F, Serman A, Sincic N (2007). The impact of 5-azacytidine on placental weight, glycoprotein pattern and proliferating cell nuclear antigen expression in rat placenta.. Placenta.

[r36] Simon JA, Kingston RE (2009). Mechanisms of polycomb gene silencing: knowns and unknowns.. Nat Rev Mol Cell Biol.

[r37] Sinclair KD, Allegrucci C, Singh R, Gardner DS, Sebastian S, Bispham J (2007). DNA methylation, insulin resistance, and blood pressure in offspring determined by maternal periconceptional B vitamin and methionine status.. Proc Natl Acad Sci USA.

[r38] Sood R, Zehnder JL, Druzin ML, Brown PO (2006). Gene expression patterns in human placenta.. Proc Natl Acad Sci USA.

[r39] Squazzo SL, O’Geen H, Komashko VM, Krig SR, Jin VX, Jang SW (2006). Suz12 binds to silenced regions of the genome in a cell-type-specific manner.. Genome Res.

[r40] Subramanian A, Tamayo P, Mootha VK, Mukherjee S, Ebert BL, Gillette MA (2005). Gene set enrichment analysis: a knowledge-based approach for interpreting genome-wide expression profiles.. Proc Natl Acad Sci USA.

[r41] Takai D, Jones PA (2002). Comprehensive analysis of CpG islands in human chromosomes 21 and 22.. Proc Natl Acad Sci USA.

[r42] Weisenberger DJ, Campan M, Long TI, Kim M, Woods C, Fiala E (2005). Analysis of repetitive element DNA methylation by MethyLight.. Nucleic Acids Res.

[r43] Wilhelm CS, Kelsey KT, Butler R, Plaza S, Gagne L, Zens MS (2010). Implications of LINE1 methylation for bladder cancer risk in women.. Clin Cancer Res.

[r44] Wolff EM, Chihara Y, Pan F, Weisenberger DJ, Siegmund KD, Sugano K (2010). Unique DNA methylation patterns distinguish noninvasive and invasive urothelial cancers and establish an epigenetic field defect in premalignant tissue.. Cancer Res.

[r45] Zamudio N, Bourc’his D. (2010). Transposable elements in the mammalian germline: a comfortable niche or a deadly trap?. Heredity.

