# Supplementary material for: In Utero Exposures, Infant Growth, and DNA Methylation of Repetitive Elements and Developmentally Related Genes in Human Placenta
Source: Environ Health Perspect. 2011 Oct 17;120(2):296–302. doi: 10.1289/ehp.1103927 (PMC3279448; doi:10.1289/ehp.1103927)
Supplement: (4.3 MB) PDF [file ehp.1103927.s001.pdf]

Supplemental Materials:

In Utero Exposures, Infant Growth, and DNA Methylation of Repetitive Element and Developmentally Related Genes in Human Placenta

C.S. Wilhelm-Benartzi, E.A. Houseman, M.A. Maccani, G.M. Poage, D.C. Koestler, S.M. Langevin, L.A. Gagne, C. Banister, J.F. Padbury, C.J. Marsit

Table of Contents:

1) Supplemental Materials, Table 1……………………………..……..………………..pg. 2
2) Supplemental Materials, Table 2……………………………..……..………………..pg. 3
3) Supplemental Materials, Figure 1 ………………………………………………… pg. 4
4) Supplemental Materials, Figure 2 ………………………………………………… pg. 5
5) Supplemental Materials, Supplementary Text 1……………..…………………...… pg. 6
6) Supplemental Materials, Supplementary Text 2……………..…………………...… pg. 7
7) Supplemental Materials, References………..……………..…………………...… pg. 8

Table 1: Population characteristics by birthweight status

Characteristic	Total population (N=380)	SGA n=62 (16.3%)	AGA n=304 (80.0%)	LGA n=14 (3.7%)	p-value
Birthweight percentile, mean +/- SD	39.3+/- 28.6	5.2 +/- 2.5	43.7 +/- 24.9	95.8 +/- 3.2	NA
Maternal age, mean +/- SD	28.1+/- 5.7	26.7 +/- 5.5	28.4 +/- 5.8	28.6 +/- 5.3	0.11
Maternal BMI before pregnancy, mean +/- SD	25.9 +/- 5.3	25.3 +/- 5.1	26.0 +/- 5.4	24.7 +/- 2.8	0.61
Gestation_weeks, mean +/- SD	38.8 +/- 1.1	38.5 +/- 1.1	38.9 +/- 1.1	38.5 +/- 0.9	0.82
Mean LINE1, mean +/- SD	51.7 +/- 4.6	50.9 +/- 4.2	51.9 +/- 4.8	51.9 +/- 3.0	<0.0001
Mean Alu Yb8, mean +/- SD	65.0 +/- 3.3	64.4 +/- 4.0	65.0 +/- 3.1	66.6 +/- 2.3	<0.0001
Array-based methylation, mean +/- SD* c.	0.24 +/- 0.01	0.24 +/- 0.01	0.24 +/- 0.02	0.23 +/- 0.01	0.59
Ethnicity mother, n (%)					
Non-Caucasian	163 (42.9)	37 (59.7)	122 (40.1)	4 (28.6)	
Caucasian	217 (57.1)	25 (40.3)	182 (59.9)	10 (71.4)	0.01
Infant gender, n (%)					
Female	190 (50.0)	35 (56.5)	149 (49.0)	6 (42.9)	
Male	190 (50.0)	27 (43.5)	155 (51.0)	8 (57.1)	0.49
Tobacco use in pregnancy, n (%)					
No	343 (90.3)	51 (82.3)	278 (91.4)	14 (100)	
Yes	36 (9.7)	11 (17.7)	25 (8.6)	0 (0)	0.03
Alcohol use in pregnancy, n (%)					
No	377 (99.2)	61 (98.4)	302 (99.3)	14 (100)	
Yes	3 (0.8)	1 (1.6)	2 (0.7)	0 (0)	0.70
Prenatal vitamin use, n (%)					
No	66 (17.4)	8 (12.9)	56 (18.4)	2 (14.3)	
Yes	314 (82.6)	54 (87.1)	248 (81.6)	12 (85.7)	0.55

a. NA=Not available, Abbreviations: SD=standard deviation, BMI= body mass index, SGA/AGA/LGA=small/appropriate/large for gestational age, LINE=long interspersed nuclear element

b. p-values were obtained by Kruskal Wallis and Chi square tests for SGA versus AGA versus LGA

c.This applies to a subsample (n=184) of our population where n=135 were AGA, n=43 were SGA and n=6 were LGA

Table 2: Distribution of CpG loci by bioinformatically-derived class.

Genomic Attributes	n_{loci}	Percent Total
None	1638	6.184%
TFBS	4436	16.748%
MIR	99	0.374%
MIR | TFBS	184	0.695%
ALU	68	0.257%
ALU | TFBS	79	0.298%
LINE2	88	0.332%
LINE2 | TFBS	103	0.389%
LINE2 | MIR | TFBS	1	0.004%
LINE-1	74	0.279%
LINE-1 | TFBS	48	0.181%
PcG	2347	8.861%
PcG | TFBS	12958	48.924%
PcG | MIR	71	0.268%
PcG | MIR | TFBS	253	0.955%
PcG | ALU	121	0.457%
PcG | ALU | TFBS	173	0.653%
PcG | LINE2	33	0.125%
PcG| LINE2 | TFBS	130	0.491%
PcG | LINE-1	24	0.091%
PcG | LINE-1 | TFBS	35	0.132%
CGI	64	0.242%
CGI | TFBS	315	1.189%
CGI | MIR	8	0.030%
CGI | MIR | TFBS	13	0.049%
CGI | ALU	3	0.011%
CGI | ALU | TFBS	1	0.004%
CGI | LINE2	3	0.011%
CGI | LINE2 | TFBS	6	0.023%
CGI | LINE-1	2	0.008%
CGI | LINE-1 | TFBS	1	0.004%
CGI | PcG	330	1.246%
CGI | PcG | TFBS	2692	10.164%
CGI | PcG | MIR	4	0.015%
CGI | PcG | MIR | TFBS	33	0.125%
CGI | PcG | ALU	10	0.038%
CGI | PcG | ALU | TFBS	21	0.079%
CGI | PcG | LINE2	4	0.015%
CGI | PcG | LINE2 | TFBS	7	0.026%
CGI | PcG| LINE-1	5	0.019%
CGI | PcG| LINE-1 | TFBS	1	0.004%
Total	26486	100%

a. Abbreviations: CGI = CpG island; TFBS = transcription factor binding sites; PcG = polycomb group protein target gene; MIR = mammalian wide-interspersed repeat

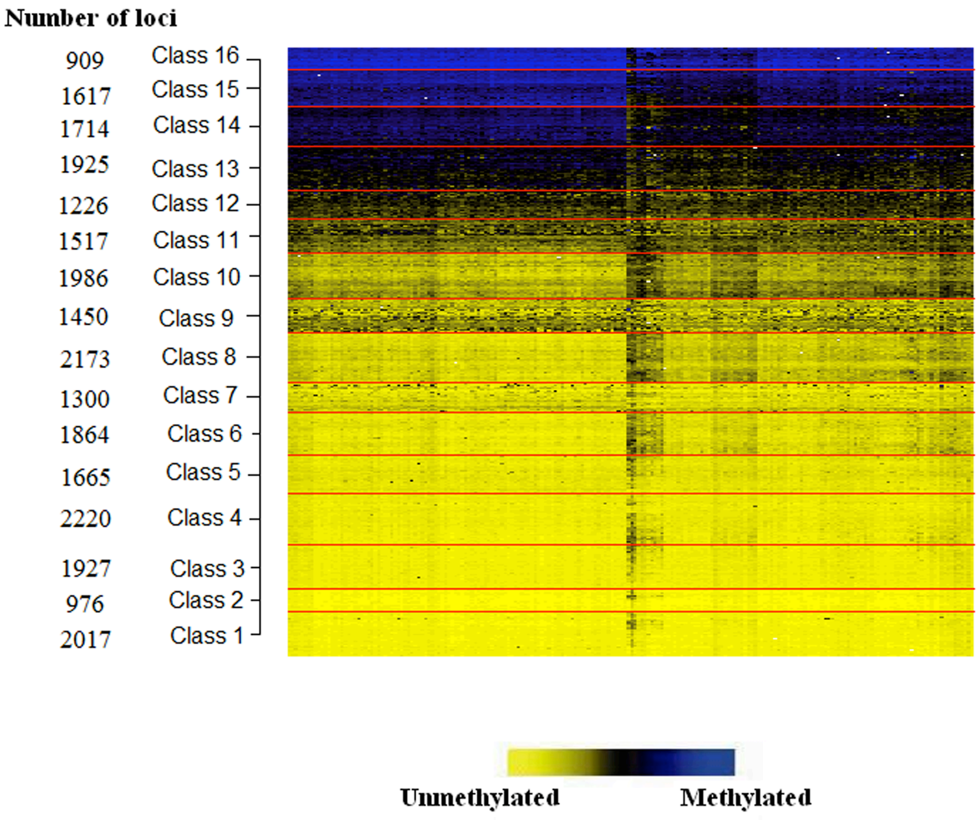

Figure 1. Methylation by RPMM class**.** The heatmap shows the average methylation (beta) by class, with the 16 RPMM-based methylation classes shown in rows and the subjects represented by columns. The number of CpG loci per RPMM class are shown on the left hand panel. The intensity of methylation is represented with yellow depicting lack of methylation and blue depicting methylation. The red lines within the heatmap denote the sixteen classes derived from the RPMM model.

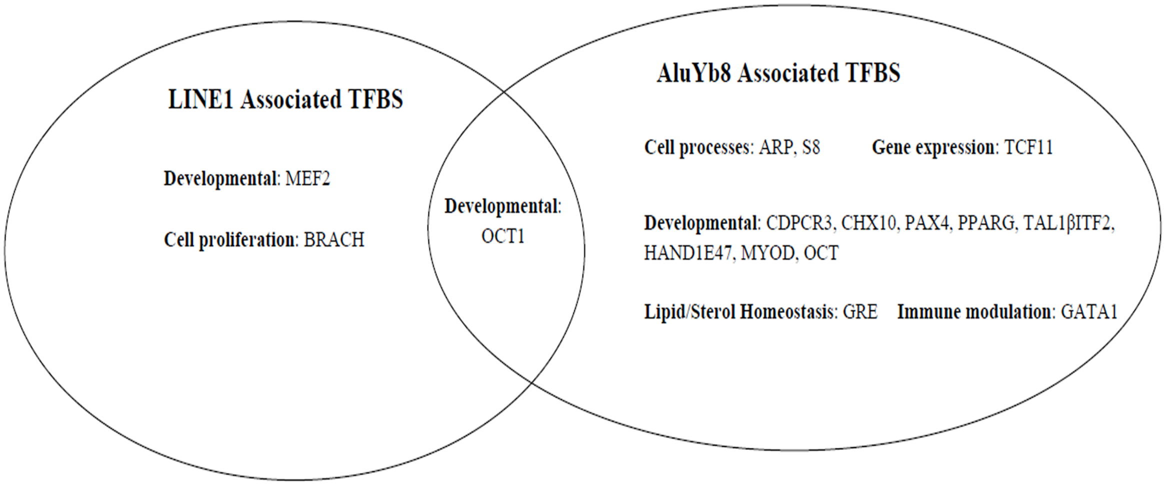

Figure 2. Over-representation of transcription factor binding sites (TFBS) within 1kb of differentially methylated loci associated with each individual repetitive element methylation marker (LINE-1 and AluYb8), grouped by functional role or family. Overlapping TFBS associated with both markers are displayed at center.

Supplementary Text 1. A series of full thickness cores of parenchymal tissue were taken from each placenta about 2 cm from the cord insertion site, free of the maternal decidua, within 2 hours after delivery. A total of 12 cores were obtained, with 3 obtained from each quadrant of the placenta, totaling approximately 1 g. Each core was approximately 1.0 cm in diameter and 1.5-2.0 cm in thickness. Each sample was rinsed and then placed in RNAlater (Applied Biosystems, Foster City, CA) and stored at 4ºC. Placental samples were snap-frozen in liquid nitrogen within 72 hours, and homogenized to powder in a cooled mortar and pestle to combine the samples from all quadrants into a single mixed sample. The subsequent powdered sample was aliquoted into 2 mL cryotubes, and was stored at -80°C until needed for nucleic acids extraction. DNA was extracted and purified from the placenta samples using the QIAamp DNA Mini Kit (Qiagen, Inc., Valencia, CA) following manufacturer's protocols. Purified DNA was quantified using a NanoDrop ND1000 spectrophotometer (ThermoFisher Scientific, Waltham, MA) and 1μg of placental DNA per sample was bisulfite modified using the EZ DNA Methylation Kit D5008 (Zymo Research, Orange, CA).

Supplementary Text 2. Performance assessment of recursively partitioned mixture model (RPMM) versus hierarchical clustering for classification of high-density DNA methylation data.

Although metric (nonparametric) hierarchical clustering is a well-characterized method, it does not scale to tens of thousands of cases; consequently, we used a Recursively Partitioned Mixture Model (RPMM), a hierarchical mixture-model algorithm described by (Houseman et al. 2008) and implemented in the R library RPMM (http://cran.r-project.org/web/packages/RPMM/index.html). Average betas for 26,486 autosomal CpG loci were clustered via RPMM according to their absolute variation of 91 beta values, as described below. Thus, the CpGs were hierarchically clustered based on their pattern of absolute variation. The resulting hierarchy of classes was pruned to 4 binary levels, resulting in 2^4 = 16 classes of CpGs. Note that we compared the consistency of RPMM clustering to that of metric clustering (using Euclidean distance with Ward's linkage) by pairwise analysis of 100 resampling experiments. We sampled 1000 probe sets at a time for each experiment, and on a pairwise basis between sampling runs, used the adjusted Rand index (Rand 1971) to compare the consistency of the clustering of CpGs that were sampled in both runs (i.e. the intersection of the sampled CpGs between two experiments). Thus the mean adjusted Rand index was computed by averaging 4950 unique pairs of experiments; standard errors were computed from the approximate sampling distribution obtained by bootstrapping the 100 individual experiments and averaging the resulting pairwise comparisons. Mean adjusted Rand index for RPMM was 0.623 (sd = 0.006) and for metric hierarchical clustering it was 0.564 (sd=0.004). Thus, RPMM also appeared to provide more consistent clustering than the more common nonparametric approach.

References

Houseman EA, Christensen BC, Yeh RF, Marsit CJ, Karagas MR, Wrensch M, et al. 2008. Model-based clustering of DNA methylation array data: a recursive-partitioning algorithm for high-dimensional data arising as a mixture of beta distributions. BMC Bioinformatics 9:365.

Rand WM. 1971. Objective criteria for the evaluation of clustering methods. JASA 66: 846–850.
